# Cognitive Impairment in Patients with Severe COPD: A Cross-Sectional Study

**DOI:** 10.3390/jcm14197122

**Published:** 2025-10-09

**Authors:** Kristina Kock Hansen, Ingeborg Farver-Vestergaard, Hanne Irene Jensen, Anders Løkke, Ole Hilberg

**Affiliations:** 1 Department of Medicine, University Hospital of Southern Denmark, Lillebaelt Hospital, Beriderbakken 4, 7100 Vejle, Denmark; kristina.kock.hansen2@rsyd.dk (K.K.H.); anders.lokke@rsyd.dk (A.L.); ole.hilberg@rsyd.dk (O.H.); 2 Department of Regional Health Research, University of Southern Denmark, J. B. Winsløws Vej 19, 3, 5000 Odense C, Denmark; hanne.irene.jensen@rsyd.dk; 3 Research Center for Integrated Healthcare Region of Southern Denmark, 6200 Aabenraa, Denmark; 4 Department of Anaesthesiology and Intensive Care, University Hospital of Southern Denmark, Lillebaelt Hospital, Beriderbakken 4, 7100 Vejle, Denmark

**Keywords:** chronic obstructive pulmonary disease, cognitive impairment, Montreal cognitive assessment, Continuous Reaction Time test, driving simulator, functional tests

## Abstract

**Background/Objectives**: Cognitive impairment (CI) in patients with chronic obstructive pulmonary disease (COPD) has been associated with reduced physical activity and decreased adherence to inhalation therapy. The primary aim of this study is to examine the prevalence of CI in patients with severe COPD and secondly compare outcomes with non-COPD comparators. **Methods**: Patients with severe COPD defined as forced expiratory volume in first second (FEV_1_) <50% were recruited between January 2021 to January 2023 along with non-COPD comparators. CI was defined as a MoCA score < 26, adding one point for participants with ≤12 years of education. Additionally, two functional cognitive tests were included: the Continuous Reaction Time test (CRT) and a driving simulator. **Results**: Eighty patients with COPD (mean age 64 years) and 22 non-COPD comparators (mean age 61 years) participated. CI was identified in 32 patients with COPD (40%) and six non-COPD comparators (27%) with a 0.87 non-significant difference (95% CI: −0.15–1.88). The functional tests showed a 0.267 difference in CRT index (95% CI: 0.023–0.511) and a 0.056 difference in standard deviation from center of the road (95% CI: 0.002–0.11) revealing a significantly poorer performance in functional tests among patients compared to non-COPD comparators. Nineteen patients with COPD and one non-COPD comparator failed the driving test (*p* = 0.04). **Conclusions**: CI was found in 40% of patients with severe COPD based on MoCA score. While MoCA score did not differ between the two groups, functional tests demonstrated significantly reduced abilities in patients compared with non-COPD comparators.

## 1. Introduction

Patients with chronic obstructive pulmonary disease (COPD) exhibit a higher frequency of comorbidities compared to the general population. Cardiovascular disease, type 2 diabetes, lung cancer, anxiety and depression as well as cognitive impairment (CI) are some of the common comorbidities in patients with COPD [1,2,3]. Obstructive sleep apnea (OSA) is also prevalent and associated with CI [4].

CI means that abilities such as perception, memory, attention, motoric skills, as well as motoric strength, speed and coordination are reduced [3,5]. In patients with COPD, CI affects everyday functioning, leading to impaired quality of life and poor compliance with medication, oxygen therapy and behavioral interventions, thereby increasing the risk of exacerbations [6]. Previous studies in COPD have shown that the risk of CI increases with age [7]. There is disagreement on whether sex is associated with CI [8,9], while low education is a known predictor. The precise etiological link between COPD and CI remains unclear as various factors, characteristics and comorbidities may be associated with CI in COPD [2].

Prevalence estimates of CI in individuals with COPD vary widely, with reported rates ranging from 10.4% to 77% [10,11]. A meta-analysis found an increased risk of CI among patients with COPD compared to non-COPD controls (OR = 1.72, *p* = 0.01) across five studies [12]. However, several studies examining COPD and cognitive function have relied on the Mini-Mental State Examination (MMSE), which has been demonstrated to be less sensitive and specific compared to the Montreal Cognitive Assessment (MoCA) screening tool [13]. Beyond cognitive screening tools such as MoCA, functional tests offer insight into how CI affects daily life. The driving simulator is an internationally recognized method for assessing perception and attention under realistic conditions [14]. Similarly, the Continuous Reaction Time (CRT) test is a quick and reliable tool for measuring sustained attention, stability, and reaction time [15]. Normally, the CRT test is used in screening for minimal hepatic encephalopathy [16] but has also been used in the examination of cognitive function in other patient groups, for example, in patients with cancer [17]. Together, these tests provide objective measures that complement traditional screening.

Nevertheless, many of the observational studies examining CI with MoCA are limited by small sample sizes, variations in cognitive assessment tools, heterogeneous sample populations with sparse descriptions of clinical characteristics and inconsistent cut off values for MoCA score [6,12].

On this background, the primary aim of our study was to examine the prevalence of CI in patients with severe COPD compared with non-COPD comparators using the MoCA screening tool. We hypothesized that patients with severe COPD would demonstrate a higher prevalence of CI than non-COPD comparators. Secondary objectives were to evaluate patient–control differences in functional cognitive performance, as measured by the CRT test and a driving simulator.

## 2. Materials and Methods

In this cross-sectional study, patients with severe COPD from the Outpatient Clinic of Respiratory Diseases at Lillebaelt Hospital, Vejle, Denmark, and non-COPD comparators were recruited in the period from January 2021 to January 2023.

### 2.1. Study Population

Patients diagnosed with severe or very severe COPD, characterized by a forced expiratory volume in one second (FEV_1_) of less than 50% of the predicted value according to the GOLD 2019 criteria [18], were considered eligible for inclusion in the study. Eligible patients were between 40 and 75 years old, had a valid driver’s license, and no COPD exacerbations within the previous three weeks. Participants in the non-COPD comparison group consisted of family members and friends of the patients as well as volunteers from the general population, who responded to advertisements on social media.

Patients and non-COPD comparators were excluded from the study if they (1) lacked the ability to operate a driving simulator (physically disabled), (2) required oxygen therapy, (3) had a history of OSA, (4) had high alcohol consumption (>21 units per week for men, >14 units per week for women), (5) had uncorrected sight or hearing reduction, (6) had serious uncontrolled comorbidities or a history of stroke or brain injury, or (7) did not read or understand Danish.

Patients without a prior OSA diagnosis were included regardless of their actual OSA status at the time of enrollment. The exclusion criteria were not influenced by de novo identification. Participants using oxygen therapy or treatment for OSA were excluded as this could potentially impact the results. Excessive alcohol consumption and those with impaired vision or hearing were also excluded, as these factors could potentially undermine the accuracy and reliability of the cognitive tests.

Written informed consent was obtained from all participants prior to data collection. The study was approved by the Danish Data Protection Agency and the Regional Committee on Health Research Ethics for Southern Denmark (S-20190082). The study was registered at Open Patient data Explorative Network (OPEN), Odense University Hospital (OP_1159) and at www.ClinicalTrials.gov (NCT04458038) prior to participant enrollment, and was performed in accordance with the Declaration of Helsinki.

### 2.2. Data Collection

Data collection was performed by KKH. Participants underwent examinations in a quiet, disturbance-free environment. The study protocol included the completion of questionnaires followed by clinical and cognitive evaluations, with the entire session lasting approximately 2.5 to 3 h. In order to minimize the potential impact of fatigue on cognitive performance, cognitive tests were conducted as the initial step.

### 2.3. Questionnaires

Participants provided information about their age, sex, cohabitation status, education level, working status, body mass index (BMI), smoking history and completed the modified Medical Research Council breathlessness scale (mMRC) [19], the COPD Assessment Test (CAT) [20] and the Hospital Anxiety and Depression Scale (HADS) [21]. Information on comorbidities, medication, and exacerbation history was collected from medical records.

### 2.4. Clinical Assessment

The following parameters were assessed: blood pressure, pulse, oral temperature, exercise capacity (using the 6-min walk test (6MWT) [22], arterial partial pressure of oxygen (PaO_2_), carbon dioxide (PaCO_2_) and oxygen saturation (SaO_2_), forced vital capacity (FVC) (L), FVC% predicted, FEV_1_ (L), FEV_1_% predicted, and FEV_1_/FVC.

Respiratory effort, nasal airflow, pulse, and oxygen saturation during sleep were measured with an at-home CardioRespiratory Monitor (CRM), NOX T3TM (ResMed, Maribo, Denmark) [23], including Apnea–Hypopnea Index (AHI) and percentage of sleep with oxygen saturation < 90% (T90). Participants with T90 > 30% were registered as having nocturnal desaturation (ND) [24]. Patients with an AHI ≥ 5 and symptoms of OSA (i.e., headache, daytime weariness, snoring or poor sleep quality) were diagnosed with OSA. The CRM was provided to the participants, along with guidance for utilizing it at home that same night and returning it the following day.

### 2.5. Cognitive Assessment

Participants were categorized based on their total MoCA score, with a score of <26 indicating CI and a score ≥ 26 indicating no CI. One additional point was added for participants with ≤12 years of education to account for the potential confounding effect of educational attainment. A cut-off value of 26 offers the optimal balance between sensitivity and specificity [25]. Five selected MoCA domains were assessed: visuospatial ability, executive functioning, attention, language, and short-term memory through delayed recall.

Furthermore, a 20-min driving simulator test assessing perception and attention was performed using an Archimedes A5000 RISC computer [14]. The participants were placed in front of the computer screen. The computer simulated a horizon in the middle of the screen as well as a road and the front of a car in the bottom half of the screen. The participants were encouraged to keep the virtual vehicle on the road, using a steering wheel in front of them. If patients (before the 20 min were up) drove off-road for a certain period of time, the test stopped automatically. It was recorded as a test failure and the driving time was registered. Participants’ abilities to operate the driving simulator were evaluated, expressed as the standard deviation (SD) of the car’s distance from the center of the road and as the ability to press a button on the steering wheel, whenever the number “2” appeared on the screen (average response time in seconds).

In addition, the participants completed the Continuous Reaction Time (CRT) test, and the CRT index measures the stability in a person’s ability to react [15]. The CRT measurement procedure typically spans approximately 10 min. Utilizing EKHO software version 18.1.6598 (Bitmatic, Aarhus, Denmark) on a computer, auditory stimuli were generated. A total of 100 stimuli were randomly delivered through headphones. The participants were required to promptly press a handheld thumb-trigger button when a sound was detected [16]. A CRT index > 1.9 is considered normal [15].

### 2.6. Statistics

Descriptive statistics were applied, and the distribution of continuous measures was evaluated for normality. The residuals were examined for normality and homogeneity of variance.

For data following a normal distribution, comparisons between groups were analyzed with independent samples *t*-tests. Outcomes that deviated from normality were examined using the Mann–Whitney U test. Chi-squared was used for categorical data, and analyses of variance (ANOVA) were used when comparing normally distributed data in more than two sub-groups. The Kruskal–Wallis test was used as the non-parametric alternative.

To explore potential links between cognitive outcomes and pulmonary function measured by FEV_1_, a multivariable regression analysis was conducted, adjusting for covariates known to be associated with cognitive function (age, sex, education level, PaO_2_, anxiety and OSA (yes/no)) [11,26,27]. Independent variables lacking normal distribution, such as SD from center of the road and mean response time, were log-transformed to approximate normality. To avoid multicollinearity, highly correlated independent variables were not included simultaneously in the analyses; for example, FEV_1_ and FEV_1_/FVC or depression and anxiety.

All statistical tests were conducted as two-sided, and results were regarded as significant when *p* < 0.05. Estimates are reported together with 95% confidence intervals. Based on relevant studies [28,29], the sample size calculation indicated that, with a mean MoCA score of 26, a power of 0.9, a difference of 2 points, a SD of 2, an alpha level of 0.05 and a patient–comparator ratio of 5:1, a minimum of 65 patients and 13 non-COPD comparators were required. Data was registered into the secure web application Research Electronic Data Capture (REDCap), and Stata version 17.0 was used for statistical analysis.

## 3. Results

**Figure 1:**
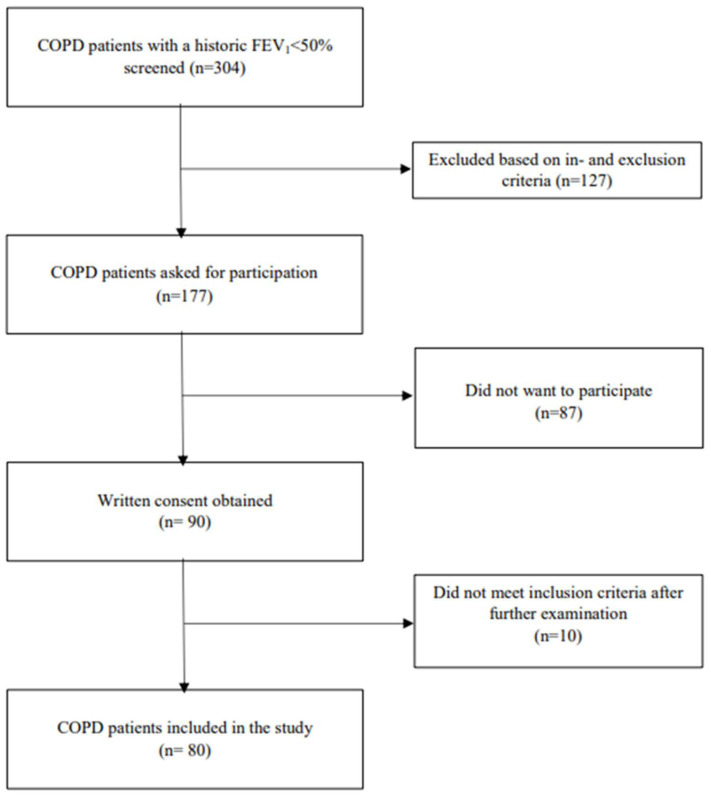
Study flowchart of the inclusion process. Modified from Figure 1 in [30]. Abbreviations: FEV_1_, forced expiratory volume in 1 s, % of predicted; OSA, obstructive sleep apnea.

The study included 80 out of 177 eligible patients with COPD (inclusion rate = 45%). Study flowchart (Figure 1), reproduced from the study by Hansen et al. [30], illustrates the inclusion process. The main reason for not participating was that patients declined. Twenty-two non-COPD comparators were included. A total of 58 patients (73%) were spirometrically classified as GOLD 3, and 22 patients (27%) were classified as GOLD 4.

### 3.1. Participant Characteristics

Patients and non-COPD comparators differed in a number of sociodemographic and disease-related factors (Table 1).

We found a significant difference in the use of medicine classified under the central nervous system, N, in the Anatomical Therapeutic Chemical (ATC) classification system. However, no significant differences were observed in the use of opioids, antiepileptics, antipsychotics, or antidepressant. The vast majority of this difference is due to a significantly greater consumption of Paracetamol in patients with COPD compared to non-COPD comparators.

### 3.2. Cognitive Impairment

A total of 32 patients (40%) had CI based on MoCA score compared to six non-COPD comparators (27%), with a non-significant difference at 0.87 (95% CI: −0.15–1.88) (Table 2). Findings revealed a significantly poorer performance in functional tests among patients compared to non-COPD comparators.

When comparing the continuous CRT index, patients had a significantly lower CRT index, indicating a reduced stability in reaction time than non-COPD comparators (*p* = 0.03) with a 0.267 difference in CRT index (95% CI: 0.023–0.511).

Patients had significantly higher SD from center of the road than non-COPD comparators (*p* = 0.04) with a 0.056 difference in standard deviation from center of the road (95% CI: 0.002–0.11). We looked at all participants, including those who did not complete the test.

Nineteen patients with COPD and one non-COPD comparator failed the driving test (*p* = 0.04).

### 3.3. Characteristics of Patients with a MoCA Score < 26 and ≥26

In a subgroup analysis, patients with a MoCA score < 26 and ≥26 were compared (Table 3).

Patients with a MoCA score < 26 were significantly older, had higher systolic blood pressure, mMRC, and CAT score, and poor driving outcomes (perception and attention). The results for 6MWT did not reach significance (*p* = 0.05). The remaining factors such as education level, PaO_2_, number of comorbidities or exacerbations were not statistically significant.

### 3.4. Non-Completion of Cognitive Tests

Another subgroup analysis, dividing patients into three groups of driving time (<10, 10–19.99 and 20 min), showed that patients who drove <10 min were significantly older, had lower exercise capacity and total MoCA score as well as higher SD from center of the road compared to patients who drove ≥10 min (Table 4).

### 3.5. Supplementary Subgroup Analysis

When comparing cognitive function between GOLD 3 and GOLD 4, no statistically significant differences were seen in MoCA score, CRT index, or in driving outcomes (Table S1).

A multiple regression analysis showed no significant association between lung function (FEV_1_) and cognitive function in patients with COPD (Tables S2 and S3). Even after adjustment for age, sex, education level, PaO_2_, anxiety, and OSA, no statistically significant associations were found.

### 3.6. Missing Data

One patient did not perform the driving test because more than one year had passed since the patient had last driven. Five patients did not complete the 6MWT due to leg pain or lack of muscle strength. In 12 patients, the arterial blood-gas test was forgotten or refused by the patient. Three non-COPD comparators had the arterial blood-gas test taken.

## 4. Discussion

In our study, we found CI in 40% of patients with severe COPD based on MoCA score. Additionally, results from both functional tests (CRT and driving simulator) indicate that patients with severe COPD performed worse than non-COPD comparators. Significantly, more patients did not complete the driving test and had a lower CRT index and a higher SD from center of the road.

### 4.1. Cognitive Impairment

In our study, only patients with severe and very severe COPD were included. In the literature [28], CI is associated with disease severity. Therefore, we may have found a higher prevalence of CI in our study compared to the overall COPD population.

A review by Ward et al. [32] examined CI in different general populations in Asia, Europe and United States and found the prevalence of CI ranging from 3% to 42% in individuals aged ≥55 years with a median prevalence of 26.4%, similar to our non-COPD comparators.

Studies with similar cut off value in MoCA score have examined CI in stable COPD and have yielded prevalence rates ranging from 27% to 63% [33,34]. The 40% CI prevalence of our study aligns closely with the two studies by Andrianopoulos et al. from 2018 [35] and 2021 [36], with prevalences of 40% and 42%, respectively. However, the two studies were not fully comparable to ours in relation to GOLD groups, with a mean FEV_1_ of 51% and 47.6% compared to 35% in our study.

To our knowledge, no studies have examined cognitive function in patients with COPD studying patients in GOLD 3 and 4 alone, like in our study. However, three comparable studies [28,37,38] have divided their patients into GOLD groups and measured mean MoCA scores or CI prevalence. One of the three studies by Dag et al. [38] found a prevalence of CI in GOLD 3 and 4 of 37.5% and 25%, respectively. We found a CI prevalence of 41% and 36% in GOLD 3 and 4, which is equal to the findings in the study by Dag et al. The variation in prevalence of CI and mean MoCA score between studies may be due to, e.g., differences in age, sample sizes, and disease severity of the included patients.

The difference in CI in patients and non-COPD comparators, based on MoCA score, did not reach statistical significance in our study. One explanation could be that patients who chose to participate in our study with a comprehensive test battery may have more resources and be less affected by their disease, compared to patients with severe and very severe COPD in general.

The lack of significance may also be explained by power limitations even though the power calculation was carried out based on existing research [28,29].

### 4.2. Characteristics of Patients with Cognitive Impairment

In our study, patients with CI based on MoCA score were older and had higher systolic blood pressure, mMRC, and CAT score compared to patients without CI. It is well known that age is associated with cognitive impairment. A study by Tudorache et al. [7] shows that cognitive function decline with age in patients with COPD.

According to some studies [6,39], CI is associated with COPD severity, especially FEV_1_ and PaO_2_. However, another study indicates no association between lung function and CI [7]. In our study, we did not find any associations between FEV_1_ and CI; maybe because we only included patients with severe and very severe COPD and not patients with mild and moderate COPD. However, based on mMRC and CAT score, our study did show an association between CI and health impact.

### 4.3. Assessment of Cognitive Function

In this study, we found that patients who drove <10 min had a significantly lower mean MoCA score compared to patients driving ≥10 min. Additionally, we found that more patients failed the driving test and had a higher SD from the center of the road than non-COPD comparators. Therefore, the driving simulator appears to be a useful tool for functional evaluation of CI. However, there is currently no consensus on the most suitable methods for assessing cognition in patients with COPD. No specific cognitive tests are recommended for this population, and existing tools may not be equally valid for detecting CI in this context. Future studies should consider adopting a standardized neuropsychological battery to enhance consistency and comparability across findings. In clinical settings, it is equally important to carefully evaluate which cognitive tests are most appropriate for use—whether the goal is to assess overall cognitive function or to examine specific cognitive domains.

Additionally, there is a lack of consensus regarding the operational definition of CI, including variability in cut-off scores and the number of administered neuropsychological measures. This lack of agreement can introduce inconsistency in research findings.

### 4.4. Strengths and Limitations

A strength of our study is the limited amount of missing data. The first author (KKH) performed all the examinations, and managed and analyzed data, increasing the internal validity of the study. However, performing the examinations and analyses may also be seen as a limitation, potentially introducing information bias.

The sample size of our study may have been a limitation. We did not find significant differences in MOCA score between patients and non-COPD comparators. A small sample size increases the risk of both type 1 and type 2 errors. As a result, we may have mistakenly rejected the null hypothesis of no difference, even if one actually existed. A larger sample size might have revealed a significant difference in MOCA score. However, recruiting patients with severe COPD is challenging, and our sample size of 80 patients is relatively large compared to similar studies.

We did not apply type 1 error control procedures such as Bonferroni or FDR corrections. The rationale was that applying such adjustments in the context of our limited sample size could substantially increase the risk of type 2 errors and potentially obscure clinically relevant differences. We agree that this represents a limitation, and we emphasize that the exploratory design should be interpreted with caution and that the findings are primarily hypothesis-generating.

Another limitation of our study concerns the use of the MoCA correction for education. While we applied the recommended adjustment of adding one point for participants with ≤12 years of education [25], it is important to acknowledge that this may influence prevalence estimates of CI. The correction is particularly relevant in the context of the well-documented educational gradient across demographic groups. It is widely recognized that individuals with COPD generally have a lower educational level compared to the general population, making the adjustment particularly relevant in this study [40].

The patient inclusion rate of 45% is also a limitation. The cognitive function of patients who declined to participate is unknown, but their age and FEV_1_ were comparable to those included. The small sample size of the comparison group is a limitation. Furthermore, a considerable part of the non-COPD comparators was relatives to the patients and therefore might share common risk factors, including nutritional and environmental exposures. This could potentially make them less representative of the general population and affect the generalizability and could contribute to the absence of a significant difference in CI between patients and non-COPD comparators. However, patients and comparators differed in several socioeconomic and disease-related factors (Table 1).

Excluding individuals treated with oxygen or without a valid driver’s license reduces the generalizability and may have introduced bias in favor of higher cognitive function. Finally, several factors may influence CI and may lead to residual confounding.

### 4.5. Implication to Clinical Practice

Structured cognitive screening, such as MoCA with correction for education, may have a role in the future standard care of patients with severe COPD, particularly when integrated with pulmonary rehabilitation and patient education concerning inhaled medication. However, as patient–control differences in MoCA score were not statistically significant, recommendations for routine screening should be made with caution. Further research is needed to clarify whether MoCA or a standardized cognitive test battery is most appropriate for COPD clinics and for which clinical purposes. Future research should include longitudinal studies to clarify the course of cognitive decline and identify key determinants such as nocturnal hypoxemia, obstructive sleep apnea (OSA), and systemic inflammation. Furthermore, intervention trials are needed to evaluate the effectiveness of rehabilitation and OSA/ND management, with particular emphasis on cognitive outcomes.

## 5. Conclusions

In our study, we found CI in 40% of patients with severe COPD based on MoCA score with a non-significant patient–control difference. In addition, results from both the CRT and the driving simulator point towards a worse performance of patients with severe COPD than non-COPD comparators, but the findings are exploratory and may be subject to bias and multiple comparisons. Therefore, the additional use of functional tests should be further explored when evaluating CI in patients with COPD.

## Figures and Tables

**Table 1:** Baseline characteristic of patients with COPD and controls.

	Patients with COPD (*n* = 80)	Controls (*n* = 22)	*p*
Age (yrs), mean (SD)	64 (7)	61 (9)	0.13
Sex (male), *n* (%)	42 (53)	7 (32)	0.09
Cohabitation, *n* (%)			
Living alone	27 (34)	1 (5)	<0.01
Living with partner	53 (66)	21 (95)	
Education level, *n* (%)			
None	17 (21)	4 (18)	<0.01
Short (2–3 yrs)	46 (58)	6 (27)	
Moderate/Long (3–6 yrs)	17 (21)	12 (55)	
Employment status, *n* (%)			
Not working	21 (26)	0 (0)	<0.01
Working	22 (28)	12 (55)	
Pensioner	37 (46)	10 (45)	
Smoking, *n* (%)			
Smoker	30 (37)	1 (5)	0.00
Former	50 (63)	9 (41)	
Never	0 (0)	12 (54)	
Body mass index (kg/m^2^), mean (SD)	25 (5)	25 (5)	0.79
Blood pressure (mmHg), mean (SD)			
Systolic	138 (15)	137 (11)	0.82
Diastolic	83 (10)	84 (11)	0.69
Pulse (beats/min), mean (SD)	83 (15)	70 (11)	0.00
Saturation (percent), mean (SD)	96 (2)	98 (1)	0.00
Temperature (Celsius), mean (SD)	36.4 (0.4)	36.3 (0.6)	0.54
mMRC (0–4), median (IQR)	2 (2–3)	0 (0–0)	0.00
Exacerbations < 1 year, *n* (%)			
None	29 (36)	-	
1	24 (30)	-	
2	13 (16)	-	
>2	14 (18)	-	
CAT score (0–40), mean (SD)	18 (7)	-	
Lung function, mean (SD)			
FEV_1_, % of predicted	35 (8)	99 (11)	0.00
FEV_1_/FVC	41.54 (9.03)	80.40 (6.65)	0.00
Arterial blood test, mean (SD)	*n* = 68	*n* = 3	
pH	7.43 (0.02)	7.42 (0.02)	0.29
PaCO_2_ (kPa)	5.15 (0.55)	5.07 (0.06)	0.26
PaO_2_ (kPa)	9.83 (1.25)	13.17 (0.61)	<0.01
Comorbidity (ICD-10), *n* (%)			
0 comorbidity	11 (14)	12 (55)	0.00
1–2 comorbidities	30 (37)	6 (27)	
>3 comorbidities	39 (49)	4 (18)	
Charlson Comorbidity Index Score, median (IQR)	1 (1–2)	0 (0–0)	0.00
HADS—Anxiety (0–21), mean (SD)	5 (4)	2 (2)	0.00
HADS—Depression (0–21), mean (SD)	3 (3)	1 (1)	0.00
Medicine (ATC system)			
N–central nervous system	24 (30)	1 (5)	0.01
N02A–opioid	6 (8)	0 (0)	0.34
N03A–antiepileptic	4 (5)	0 (0)	0.58
N05A–antipsychotic	2 (3)	0 (0)	1.00
N06A–antidepressant	9 (11)	1 (5)	0.69
6-min walk (meter), mean (SD)	382 (98) *	580 (61)	0.00
Diagnosed with OSA and/or ND, *n* (%)	56 (70)	6 (27)	0.00
AHI (numbers per hour), median (IQR)	8 (5–13)	9 (3–14)	0.72
T90 (percentage), median (IQR)	24 (7–69)	1 (0–2)	0.00

* *n* = 75. Continuous variables are specified as mean (SD) and categorical variables as number (%). mMRC, Charlson Comorbidity Index Score and sleep apnea data are not distributed normally and therefore we use median (IQR). We use a parametric unpaired *t*-test for continuous normally distributed data, and a nonparametric Mann–Whitney U-test for data not normally distributed. Using Chi-squared test for categorical data. Abbreviations: mMRC, modified Medical Research Council dyspnea scale; CAT score, COPD Assessment Test score; FEV_1_, forced expiratory volume in 1 s, % of predicted; FEV_1_/FVC, ratio between forced expiratory volume in 1 s and forced vital capacity; pH, hydrogen potential; PaCO_2_, arterial partial pressure of carbon dioxide; PaO_2_, arterial partial pressure of oxygen; ICD-10, International Statistical Classification of Disease and Related Health Problems, 10th revision; HADS, Hospital Anxiety and Depression Scale; ATC system, Anatomical Therapeutic Chemical classification system; OSA, obstructive sleep apnea; ND, nocturnal desaturation; AHI, Apnea–Hypopnea Index; T90, percentage of sleep with oxygen saturation under 90%.

**Table 2:** Outcome variables in patients with COPD and controls.

	Patients with COPD (*n* = 80)	Controls (*n* = 22)	Differences (95% CI)	*p*
MoCA score, mean (95% CI)	25.95 (25.45–26.45)	26.82 (25.91–27.72)	0.87 (−0.15–1.88)	0.09
<26, *n* (%)	32 (40)	6 (27)		0.27
26–30 (normal), *n* (%)	48 (60)	16 (73)		
Specific domains from MoCA, median (95%CI)				
Visuospatial (0–4)	2 (1–2)	2 (2–2)		0.02
Executive function (0–4)	3 (3–3)	4 (3–4)		0.07
Attention (0–6)	6 (6–6)	6 (6–6)		0.32
Language (0–5)	5 (5–5)	5 (5–5)		0.16
Short-term memory (0–5)	3 (3–4)	4 (3–4)		0.88
CRT index, mean (95% CI)	2.039 (1.884–2.194)	2.306 (2.111–2.500)	0.267 (0.023–0.511)	0.03
≤1.900, *n* (%)	38 (48)	4 (18)		0.01
>1.900, *n* (%)	42 (52)	18 (82)		
Driving time, *n* (%)	*n* = 79	*n* = 22		
<20 min	19 (24)	1 (5)		0.04
20 min	60 (76)	21 (95)		
SD from center of the road, median (95% CI)	0.365 (0.334–0.455)	0.309 (0.252–0.452)	0.056 (0.002–0.11)	0.04
Average response time (sec), median (95% CI)	2.63 (2.33–2.99)	2.6 (2.41–3.09)	0.03 (−0.49–0.56)	0.72

Continuous variables are specified as mean (CI) and categorical variables as number (%). Specific domains and driving data are not distributed normally and therefore we use median (95% CI). The 95% CI is estimated using binomial method [31]. We use a parametric unpaired *t*-test for continuous MoCA score and CRT index, and a nonparametric Mann–Whitney U-test for specific domains and the driving outcome variables. Chi-squared test for categorical MoCA score, categorical CRT index and driving time between the groups. Abbreviations: MoCA, Montreal Cognitive Assessment; 95% CI, 95% confidence interval; CRT index, Continuous Reaction Time index; SD, standard deviation.

**Table 3:** Characteristics of patients with MoCA score < 26 and MoCA score 26–30.

	Patients with CI (*n* = 32)	Patients without CI (*n* = 48)	*p*
Age (years), mean (SD)	67 (8)	62 (6)	<0.01
Sex (male), mean (SD)	15 (47)	27 (56)	0.41
Cohabitation, *n* (%)			
Living alone	9 (28)	18 (38)	0.39
Living with partner	23 (72)	30 (62)	
Education level, *n* (%)			
None	9 (28)	8 (17)	0.37
Short (2–3 yrs)	18 (56)	28 (58)	
Moderate/long (3–6 yrs)	5 (16)	12 (25)	
Employment status, *n* (%)			
Not working	7 (22)	14 (29)	<0.01
Working	3 (9)	19 (40)	
Pensioner	22 (69)	15 (31)	
Smoking, *n* (%)			
Smoker	12 (38)	18 (38)	1.00
Former	20 (62)	30 (62)	
Never	0 (0)	0 (0)	
Body mass index (kg/m^2^), mean (SD)	25 (5)	25 (5)	0.52
Blood pressure (mmHg), mean (SD)			
Systolic	142 (14)	135 (15)	0.03
Diastolic	81 (11)	84 (10)	0.30
Pulse (beats/min), mean (SD)	81 (18)	84 (13)	0.41
Saturation (%), mean (SD)	95 (2)	96 (2)	0.69
Temperature (Celsius), mean (SD)	36.4 (0.4)	36.4 (0.5)	0.98
mMRC (0–4), median (IQR)	3 (2–3)	2 (1–3)	0.02
Exacerbations < 1 year, *n* (%)			
None	12 (38)	17 (36)	0.45
1	8 (25)	16 (33)	
2	4 (12)	9 (19)	
>2	8 (25)	6 (12)	
CAT score (0–40), mean (SD)	19 (6)	16 (7)	<0.05
FEV_1_ (% of predicted), mean (SD)	36 (8)	34 (9)	0.27
PaCO_2_ (kPa), mean (SD)	5.03 (0.54) (*n* = 27)	5.23 (0.56) (*n* = 41)	0.15
PaO_2_ (kPa), mean (SD)	9.79 (1.28) (*n* = 27)	9.86 (1.25) (*n* = 41)	0.83
Number of comorbidities, *n* (%)			
0 comorbidity	2 (6)	9 (19)	0.28
1–2 comorbidities	13 (41)	17 (35)	
>3 comorbidities	17 (53)	22 (46)	
6-min walk (meter), mean (SD)	355 (102) (*n* = 30)	400 (92) (*n* = 45)	>0.05
Diagnosed with OSA and/or ND, *n* (%)	20 (63)	36 (75)	0.23
AHI (numbers per hour), median (IQR)	8 (5–15)	8 (5–12)	0.64
T90 (percentage), median (IQR)	22 (1–44)	25 (10–79)	0.15
CRT index, mean (SD)	1.947 (0.714)	2.1 (0.684)	0.34
Low (<1.9), *n* (%)	17 (53)	21 (44)	0.41
Normal (≥1.9), *n* (%)	15 (47)	27 (56)	
SD from center of the road, median (IQR)	0.509 (0.317–4.523) (*n* = 31)	0.339 (0.289–0.822)	0.04
Average time response (sec.), median (IQR)	3.71 (2.21–4.75) (*n* = 31)	2.39 (2.03–3.06)	<0.01

Continuous variables are specified as mean (SD) and categorical variables as number (%). mMRC, driving, and sleep apnea data are not normally distributed and therefore we use median (IQR). We use a parametric unpaired *t*-test for normally distributed data, a nonparametric Mann–Whitney U-test for data not normally distributed, and a Chi-squared test for categorical data. Abbreviations: MoCA, Montreal Cognitive Assessment; mMRC, modified Medical Research Council dyspnea scale; CAT score, COPD Assessment Test score; FEV_1_, forced expiratory volume in 1 s, % of predicted; PaCO_2_, arterial partial pressure of carbon dioxide; PaO_2_, arterial partial pressure of oxygen; OSA, obstructive sleep apnea; ND, nocturnal desaturation; AHI, Apnea–Hypopnea Index; T90, percentage of sleep with oxygen saturation under 90%; CRT index, Continuous Reaction Time index; SD standard deviation.

**Table 4:** Characteristics of patients with COPD distributed on driving time.

Driving Time (Minutes)	<10 (*n* = 13)	10–19.99 (*n* = 6)	20 (*n* = 60)	*p*
Age (yrs), mean (SD)	69 (5)	67 (7)	62 (7)	<0.01
Sex (male), *n* (%)	3 (23)	3 (50)	35 (58)	0.07
6-min walk (meter), mean (SD)	321 (113) *	390 (110)	397 (89) **	0.047
MoCA score, mean (SD)	25 (3)	27 (2)	26 (2)	0.04
Low (<26), *n* (%)	8 (62)	1 (17)	22 (37)	0.13
Normal (26–30), *n* (%)	5 (38)	5 (83)	38 (63)	
CRT index, mean (SD)	1.883 (0.528)	1.995 (0.450)	2.074 (0.753)	0.67
Low (<1.9), *n* (%)	4 (31)	3 (50)	31 (52)	0.39
Normal (≥1.9), *n* (%)	9 (69)	3 (50)	29 (48)	
SD from center of the road, median (IQR)	7.305 (5.791–10.332)	3.662 (2.963–4.523)	0.335 (0.289–0.432)	<0.001
Average response time (seconds), median (IQR)	3.94 (2.76–5.7)	1.99 (1.49–4.75)	2.52 (2.19–3.32)	0.093

Only sex, statistically significant covariates and outcome variables are listed in Table 4. One patient did not do the driving test. * *n* = 12, ** *n* = 56. Continuous variables are specified as mean (SD) and categorical variables as number (%). Driving outcome variables are not distributed normally and therefore we use median (IQR). Using one-way ANOVA on normally distributed continuously data and Chi-squared for categorical data, we test if there is a difference between the three groups. Using Kruskal–Wallis test for data that are not normal distributed, to examine if there is difference between the three groups. Abbreviations: MoCA, Montreal Cognitive Assessment; CRT index, Continuous Reaction Time index; SD, standard deviation.

## Data Availability

The data presented in this study are available on request from the corresponding author due to privacy reasons.

## Supplementary Materials

The following supporting information can be downloaded at: https://www.mdpi.com/article/10.3390/jcm14197122/s1, Table S1: Baseline characteristic and outcome variables—GOLD 3 and GOLD 4 patients. Table S2: Associations between lung function (FEV_1_) and cognitive function in patients with COPD, who completed the driving test. Table S3: Associations between lung function (FEV_1_) and cognitive functions in patients with COPD, including those who failed the driving test.

## Abbreviations

The following abbreviations are used in this manuscript:

AHI	Apnea Hypopnea Index
ANOVA	Analyses of variance
ATC	Anatomical Therapeutic Chemical
BMI	Body Mass Index
CAT	COPD Assessment Test
CI	Cognitive Impairment
COPD	Chronic Obstructive Pulmonary Disease
CRM	CardioRespiratory Monitor
CRT	Continuous Reaction Time test
FEV_1_	Forced expiratory volume in 1 s
FVC	Forced vital capacity
GOLD	Global initiative for chronic Obstructive Lung Disease
HADS	Hospital Anxiety and Depression Scale
IQR	Interquartile Range
mMRC	Modified Medical Research Council breathlessness
MMSE	Mini-Mental State Examination
MoCA	Montreal Cognitive Assessment
OPEN	Open Patient data Explorative Network
OSA	Obstructive Sleep Apnea
PaCO_2_	Partial pressure of carbon dioxide
PaO_2_	Partial pressure of oxygen
SaO_2_	Arterial oxygen saturation
SD	Standard deviation
T90	Percentage of sleep with oxygen saturation under 90%
6MWT	6-min walk test

## References

[1] Morley J.E. (2014). Chronic obstructive pulmonary disease: A disease of older persons. J. Am. Med. Dir. Assoc..

[2] Dodd J.W., Getov S.V., Jones P.W. (2010). Cognitive function in COPD. Eur. Respir. J..

[3] Ouellette D.R., Lavoie K.L. (2017). Recognition, diagnosis, and treatment of cognitive and psychiatric disorders in patients with COPD. Int. J. Chronic Obstr. Pulm. Dis..

[4] Wang Y., Li B., Li P., Gong T., Wu M., Fu J., Nie M., Dong Y., Hu K. (2020). Severe obstructive sleep apnea in patients with chronic obstructive pulmonary disease is associated with an increased prevalence of mild cognitive impairment. Sleep Med..

[5] Klein M., Gauggel S., Sachs G., Pohl W. (2010). Impact of chronic obstructive pulmonary disease (COPD) on attention functions. Respir. Med..

[6] Schou L., Østergaard B., Rasmussen L.S., Rydahl-Hansen S., Phanareth K. (2012). Cognitive dysfunction in patients with chronic obstructive pulmonary disease—A systematic review. Respir. Med..

[7] Tudorache E., Fildan A.P., Frandes M., Dantes E., Tofolean D.E. (2017). Aging and extrapulmonary effects of chronic obstructive pulmonary disease. Clin. Interv. Aging.

[8] Au B., Dale-McGrath S., Tierney M.C. (2017). Sex differences in the prevalence and incidence of mild cognitive impairment: A meta-analysis. Ageing Res. Rev..

[9] Tervo S., Kivipelto M., Hänninen T., Vanhanen M., Hallikainen M., Mannermaa A., Soininen H. (2004). Incidence and risk factors for mild cognitive impairment: A population-based three-year follow-up study of cognitively healthy elderly subjects. Dement. Geriatr. Cogn. Disord..

[10] Raffaele A.I., Andrea C., Claudio P., Luigi T., Domenico A., Aldo S., Orsola I., Franco R. (2006). Drawing impairment predicts mortality in severe COPD. Chest.

[11] Grant I., Heaton R.K., McSweeny A.J., Adams K.M., Timms R.M. (1982). Neuropsychologic findings in hypoxemic chronic obstructive pulmonary disease. Arch. Intern. Med..

[12] Zhang X., Cai X., Shi X., Zheng Z., Zhang A., Guo J., Fang Y. (2016). Chronic Obstructive Pulmonary Disease as a Risk Factor for Cognitive Dysfunction: A Meta-Analysis of Current Studies. J. Alzheimer’s Dis..

[13] Ciesielska N., Sokołowski R., Mazur E., Podhorecka M., Polak-Szabela A., Kędziora-Kornatowska K. (2016). Is the Montreal Cognitive Assessment (MoCA) test better suited than the Mini-Mental State Examination (MMSE) in mild cognitive impairment (MCI) detection among people aged over 60? Meta-analysis. Psychiatr. Pol..

[14] Land M., Horwood J. (1995). Which parts of the road guide steering?. Nature.

[15] Lauridsen M.M., Thiele M., Kimer N., Vilstrup H. (2013). The continuous reaction times method for diagnosing, grading, and monitoring minimal/covert hepatic encephalopathy. Metab. Brain Dis..

[16] Lauridsen M.M., Grønbæk H., Næser E.B., Leth S.T., Vilstrup H. (2012). Gender and age effects on the continuous reaction times method in volunteers and patients with cirrhosis. Metab. Brain Dis..

[17] Boelsbjerg H.B., Kurita G.P., Sjøgren P., Hansen N.V. (2022). Combining subjective and objective appraisals of cognitive dysfunction in patients with cancer: A deeper understanding of meaning and impact on suffering?. Support. Care Cancer.

[18] Singh D., Agusti A., Anzueto A., Barnes P.J., Bourbeau J., Celli B.R., Criner G.J., Frith P., Halpin D.M.G., Han M. (2019). Global Strategy for the Diagnosis, Management, and Prevention of Chronic Obstructive Lung Disease: The GOLD science committee report 2019. Eur. Respir. J..

[19] Bestall J.C., Paul E.A., Garrod R., Garnham R., Jones P.W., Wedzicha J.A. (1999). Usefulness of the Medical Research Council (MRC) dyspnoea scale as a measure of disability in patients with chronic obstructive pulmonary disease. Thorax.

[20] Jones P.W., Harding G., Berry P., Wiklund I., Chen W.H., Kline Leidy N. (2009). Development and first validation of the COPD Assessment Test. Eur. Respir. J..

[21] Zigmond A.S., Snaith R.P. (1983). The hospital anxiety and depression scale. Acta Psychiatr. Scand..

[22] Holland A.E., Spruit M.A., Troosters T., Puhan M.A., Pepin V., Saey D., McCormack M.C., Carlin B.W., Sciurba F.C., Pitta F. (2014). An official European Respiratory Society/American Thoracic Society technical standard: Field walking tests in chronic respiratory disease. Eur. Respir. J..

[23] Cairns A., Wickwire E., Schaefer E., Nyanjom D. (2014). A pilot validation study for the NOX T3(TM) portable monitor for the detection of OSA. Sleep Breath..

[24] Lewis C.A., Fergusson W., Eaton T., Zeng I., Kolbe J. (2009). Isolated nocturnal desaturation in COPD: Prevalence and impact on quality of life and sleep. Thorax.

[25] Nasreddine Z.S., Phillips N.A., Bédirian V., Charbonneau S., Whitehead V., Collin I., Cummings J.L., Chertkow H. (2005). The Montreal Cognitive Assessment, MoCA: A brief screening tool for mild cognitive impairment. J. Am. Geriatr. Soc..

[26] Eshkoor S.A., Hamid T.A., Mun C.Y., Ng C.K. (2015). Mild cognitive impairment and its management in older people. Clin. Interv. Aging.

[27] Orgeta V., Leung P., del-Pino-Casado R., Qazi A., Orrell M., Spector A.E., Methley A.M. (2022). Psychological treatments for depression and anxiety in dementia and mild cognitive impairment. Cochrane Database Syst. Rev..

[28] Yin M., Wang H., Hu X., Li X., Fei G., Yu Y. (2019). Patterns of brain structural alteration in COPD with different levels of pulmonary function impairment and its association with cognitive deficits. BMC Pulm. Med..

[29] Wong G.K.C., Mak J.S.Y., Wong A., Zheng V.Z.Y., Poon W.S., Abrigo J., Mok V.C.T. (2017). Minimum Clinically Important Difference of Montreal Cognitive Assessment in aneurysmal subarachnoid hemorrhage patients. J. Clin. Neurosci..

[30] Hansen K.K., Hilberg O., Jensen H.I., Løkke A., Farver-Vestergaard I. (2023). The Association Between Cognitive Functions and Psychological Factors in Patients with Severe COPD. Int. J. Chronic Obstr. Pulm. Dis..

[31] Hazra A. (2017). Using the confidence interval confidently. J. Thorac. Dis..

[32] Ward A., Arrighi H.M., Michels S., Cedarbaum J.M. (2012). Mild cognitive impairment: Disparity of incidence and prevalence estimates. Alzheimer’s Dement..

[33] Dulohery M.M., Schroeder D.R., Benzo R.P. (2015). Cognitive function and living situation in COPD: Is there a relationship with self-management and quality of life?. Int. J. Chronic Obstr. Pulm. Dis..

[34] Disler R.T., Spiliopoulos N., Inglis S.C., Currow D.C., Davidson P.M. (2020). Cognitive screening in chronic obstructive pulmonary disease: Patient’s perspectives. Disabil. Rehabil..

[35] Andrianopoulos V., Vogiatzis I., Gloeckl R., Bals R., Koczulla R.A., Kenn K. (2018). Cerebral oxygen availability during exercise in COPD patients with cognitive impairment. Respir. Physiol. Neurobiol..

[36] Andrianopoulos V., Gloeckl R., Schneeberger T., Jarosch I., Vogiatzis I., Hume E., Koczulla R.A., Kenn K. (2021). Benefits of pulmonary rehabilitation in COPD patients with mild cognitive impairment—A pilot study. Respir. Med..

[37] Qian H., Lin H., Li Y. (2014). Assessment of cognition and associated factors in patients with stable chronic obstructive pulmonary disease. Zhonghua Jie He He Hu Xi Za Zhi = Zhonghua Jiehe He Huxi Zazhi = Chin. J. Tuberc. Respir. Dis..

[38] Dag E., Bulcun E., Turkel Y., Ekici A., Ekici M. (2016). Factors Influencing Cognitive Function in Subjects With COPD. Respir. Care.

[39] Torres-Sánchez I., Rodríguez-Alzueta E., Cabrera-Martos I., López-Torres I., Moreno-Ramírez M.P., Valenza M.C. (2015). Cognitive impairment in COPD: A systematic review. J. Bras. Pneumol..

[40] Pallesen A.V.J., Mierau J.O., Christensen F.K., Mortensen L.H. (2024). Educational and income inequalities across diseases in Denmark: A register-based cohort study. Lancet Public Health.

