# Supplementary material for: Cognitive Impairment in Patients with Severe COPD: A Cross-Sectional Study
Source: J Clin Med. 2025 Oct 9;14(19):7122. doi: 10.3390/jcm14197122 (PMC12525021; doi:10.3390/jcm14197122)
Supplement: Supplementary file 1 [file jcm-14-07122-s001.zip › Supporting information Table S1.pdf]

Table S1 Baseline characteristic and outcome variables - GOLD 3 and GOLD 4 patients

	GOLD 3 (*n*=58)	GOLD 4 (*n*=22)	*p*
Age (yrs), mean (SD)	64 (7)	62 (8)	0.30
Sex (male), *n* (%)	29 (50)	13 (59)	0.47
Cohabitation, *n* (%)			
Living alone	20 (34)	7 (32)	0.82
Living with partner	38 (66)	15 (68)	
Education level, *n* (%)			
None	12 (21)	5 (23)	0.91
Short (2-3 yrs)	33 (57)	13 (59)	
Moderate/Long (3-6 yrs)	13 (22)	4 (18)	
Employment status, *n* (%)			
Not working	14 (24)	7 (32)	0.55
Working	15 (26)	7 (32)	
Pensioner	29 (50)	8 (36)	
Smoking, *n* (%)			
Smoker	20 (34)	10 (45)	0.37
Former	38 (66)	12 (55)	
Never	0 (0)	0 (0)	
Body Mass Index (kg/m^2), mean (SD)	26 (5)	23 (5)	0.10
Blood pressure (mmHg), mean (SD)			
Systolic	138 (16)	138 (12)	0.92
Diastolic	82 (11)	86 (10)	0.09
Pulse (beats/min), mean (SD)	81 (16)	90 (10)	<0.01
Saturation (%), mean (SD)	96 (2)	95 (2)	0.07
Temperature (Celsius), mean (SD)	36.4 (0.4)	36.5 (0.4)	0.16
mMRC (0-4), median (IQR)	2 (1-3)	3 (2-4)	0.01
Exacerbations <1 year, *n* (%)			
None	22 (38)	7 (32)	0.11
1	20 (35)	4 (18)	
2	6 (10)	7 (32)	
>2	10 (17)	4 (18)	
CAT score (0-40) , mean (SD)	16 (7)	21 (5)	<0.01
Lung function, mean (SD)			
FEV_1 (%)	39 (6)	25 (3)	<0.001
FEV_1/FVC	43.67 (8.97)	35.92 (6.55)	<0.001
Arterial blood test, mean (SD)	*n*=50	*n*=18	
pH	7.44 (0.02)	7.43 (0.03)	0.35
$PaCO_2$ (kPa)	5.07 (0.49)	5.38 (0.66)	0.08
PaO_2 (kPa)	9.91 (1.27)	9.62 (1.22)	0.40
Comorbidity, *n* (%)			
0 comorbidity	6 (10)	5 (23)	0.35
1-2 comorbidities	23 (40)	7 (32)	
>3 comorbidities	29 (50)	10 (45)	
Charlson Comorbidity Index Score, median (IQR)	1 (1-2)	1 (1-2)	0.87
6-minute walk (meter), mean (SD)	392 (99)[a]	350 (89)[b]	0.10
Diagnosed with OSA and/or ND, *n* (%)	40 (69)	16 (73)	0.74
AHI (numbers per hour), median (IQR)	8 (5-15)	8 (5-10)	0.55
T90 (percentage), median (IQR)	22 (5-65)	33 (13-78)	0.21
MoCA score, mean (SD)	26 (2)	26 (3)	0.61
<26, *n* (%)	24 (41)	8 (36)	0.68
26-30, *n* (%)	34 (59)	14 (64)	
Specific domains from MoCA, median (IQR):			
Visuospatial (0-4)	1 (1-2)	2 (1-2)	0.02
Executive function (0-4)	3 (2-4)	3 (2-4)	0.89
Attention (0-6)	6 (5-6)	6 (5-6)	0.59

Language (0-5)	5 (5-5)	5 (5-5)	0.24
Short-term memory (0-5)	4 (3-4)	3 (2-5)	0.32
CRT-index, mean (SD)	2.027 (0.692)	2.069 (0.722)	0.82
≤1.900	28 (48)	10 (45)	0.82
>1.900	30 (52)	12 (55)	
Driving time, *n* (%)	*n*=58	*n*=21	
<10 minutes	11 (19)	2 (9)	0.47
10-19.99 minutes	5 (9)	1 (5)	
20 minutes	42 (72)	18 (86)	
SD from center of the road, median (IQR)	0.335 (0.28-0.443)[c]	0.326 (0.294-0.416)[b]	0.74
Average response time (sec), median (IQR)	2.64 (2.2-3.42)[c]	2.32 (2.05-2.79)[b]	0.13

[a]*n*=57, [b]*n*=18, [c]*n*=42. Continuous variables are specified as mean (SD) and categorical variables as number (%). mMRC, Charlson Comorbidity Index Score and sleep apnea data are not normal distributed and therefore we use median (IQR). Using parametric unpaired t-test for continuous normally distributed data. Using nonparametric Mann Whitney U-test for not normal distributed data. Using Chi-squared test for categorical data.
Abbreviations: mMRC, modified Medical Research Council dyspnea scale; CAT score, COPD Assessment Test score; FEV_1, Forced Expiratory Volume in 1 second, % of predicted; FEV_1/FVC, ratio between Forced Expiratory Volume in 1 second and Forced Vital Capacity; pH, Hydrogen potential; $PaCO_2$, arterial partial pressure of carbon dioxide; PaO_2, arterial partial pressure of oxygen; OSA, obstructive sleep apnea; ND, nocturnal desaturation; AHI, Apnea-Hypopnea Index; T90, percentage of sleep with oxygen saturation under 90%; MoCA, Montreal Cognitive Assessment; CRT-index, Continuous Reaction Time Index; SD, standard deviation.
