# Supplementary material for: Cognitive Impairment in Patients with Severe COPD: A Cross-Sectional Study
Source: J Clin Med. 2025 Oct 9;14(19):7122. doi: 10.3390/jcm14197122 (PMC12525021; doi:10.3390/jcm14197122)
Supplement: Supplementary file 1 [file jcm-14-07122-s001.zip › Supporting information Table S2.pdf]

Table S2 Associations between lung function (FEV_1) and cognitive function in patients with COPD, who completed the driving test.

	Unadjusted *n*=60			Adjusted *n*=53		
	Coefficient	95% CI	*p*	Coefficient	95% CI	*p*
MoCA-score	-0.06	-0.12-0.01	0.07	-0.03	-0.11-0.04	0.33
CRT-index	0.005	-0.019-0.028	0.71	0.002	-0.027-0.031	0.87
Log SD from center of the road	0.012	-0.006-0.029	0.20	0.01	-0.007-0.026	0.24
Log average response time (sec)	0.01	-0.003-0.02	0.14	0.009	-0.004-0.022	0.19

Multiple linear regression unadjusted and adjusted. Adjusted for age, sex, education level, PaO_2, anxiety and obstructive sleep apnea. Abbreviations: MoCA, Montreal Cognitive Assessment; CRT-index, Continuous Reaction Time Index; SD, standard deviation; Log, logarithm
