# Supplementary material for: Cognitive Impairment in Patients with Severe COPD: A Cross-Sectional Study
Source: J Clin Med. 2025 Oct 9;14(19):7122. doi: 10.3390/jcm14197122 (PMC12525021; doi:10.3390/jcm14197122)
Supplement: Supplementary file 1 [file jcm-14-07122-s001.zip › Supporting information Table S3.pdf]

Table S3 Associations between lung function (FEV_1) and cognitive functions in patients with COPD, including those who failed the driving test.

	Unadjusted			Adjusted		
	Coefficient	95% CI	*p*	Coefficient	95% CI	*p*
MoCA-score, *n*=80	-0.03	-0.09-0.04	0.41	0.01[a]	-0.07-0.08	0.85
CRT-index, *n*=80	0.003	-0.016-0.022	0.74	0.002[a]	-0.022-0.026	0.86
Log SD from center of the road, *n*=78	0.033	-0.002-0.067	0.06	0.012 [b]	-0.022-0.045	0.49
Log average response time (sec), *n*=78	0.01	-0.005-0.02	0.23	-0.0004[b]	-0.01-0.01	0.96

[a]*n*=68, [b]*n*=67. Multiple linear regression unadjusted and adjusted. Adjusted for age, sex, education level, PaO_2, anxiety and sleep apnea.
Abbreviations: MoCA, Montreal Cognitive Assessment; CRT-index, Continuous Reaction Time Index; SD, standard deviation; Log, logarithm
